# Experience of a modified chest tube suture-fixation technique in uniportal thoracoscopic pulmonary resection

**DOI:** 10.1186/s12893-023-01952-5

**Published:** 2023-03-29

**Authors:** Wensong Shi, Yuzhui Hu, Cuimei Wang, Guotao Chang, Huiyu Zheng, Zhiqiang Yang, Yulun Yang, Xiaogang Zhao, Xiangnan Li

**Affiliations:** 1 Department of Thoracic Surgery, The First Affiliated Hospital of Zhengzhou University, The fifth Clinical Medical College of Henan of Chinese Medicine (Zhengzhou People’s Hospital), Zhengzhou, 450052 China; 2 Department of Geratology, Ninth People’s Hospital of Zhengzhou, Zhengzhou, 450053 China; 3 Department of Plastic Surgery, The fifth Clinical Medical College of Henan University of Chinese Medicine (Zhengzhou People’s Hospital), Zhengzhou, 450052 China; 4 Department of Thoracic Surgery, Shanghai Pulmonary Hospital Affiliated to Tongji University, Shanghai, 200433 China

**Keywords:** ERAS, Modified chest tube suture-fixation technique, Suture, U-VATS

## Abstract

**Objective:**

This study aimed to explore the feasibility and advantages of a modified chest tube suture-fixation technique in uniportal video-assisted thoracic surgery for pulmonary resection.

**Methods:**

A retrospective analysis was conducted on 116 patients who underwent uniportal video-assisted thoracic surgery (U-VATS) for lung diseases in Zhengzhou People’s Hospital between October 2019 and October 2021. Patients were stratified into two groups based on the applied suture-fixation methods, i.e., 72 patients in the active group and 44 patients in the control group. The two groups were subsequently compared in the terms of gender, age, operation method, indwelling time of chest tube, postoperative pain score, chest tube removal time, wound healing grade, length of hospital stay, incision healing grade, and patient satisfaction.

**Results:**

There was no significant difference between the two groups in terms of gender, age, operation method, indwelling time of chest tube, postoperative pain score, and length of hospital stay (P = 0.167, 0.185, 0.085, 0.051, 0.927, and 0.362, respectively). However, the chest tube removal time, incision healing grade, and incision scar satisfaction in the active group were significantly better compared with those of the control group (*P* = < 0.001, 0.033, and < 0.001, respectively).

**Conclusion:**

In summary, the new suture-fixation approach can minimize the number of stitches, and time necessary for chest tube removal process, and avoid the pain experienced when removing the drainage tube. This method is more feasible, has better incision conditions, and provides a convenient tube removal, making it more suitable to patients.

**Supplementary Information:**

The online version contains supplementary material available at 10.1186/s12893-023-01952-5.

## Introduction

In recent years, video-assisted thoracic surgery (VATS) has evolved as a minimally invasive surgery for diseases in the field of thoracic surgery [1, 2]. The technique has many advantages, including causing less trauma to the chest wall, early remission of postoperative pain, less bleeding, improved cardiopulmonary function, lower complication rates, and rapid recovery. Moreover, patients who start early adjuvant chemotherapy show better immunological responses and stress hormone responses [3, 4], reduced length of hospital stay, and chest tube indwelling time. VATS allows faster recovery of patients to their normal life and work. Notably, concerns regarding less pain and improved cosmesis fueled the evolution of uniportal access. The uniportal video-assisted thoracic surgery (U-VATS) technique was discovered following the modification and development of the two-port and three-port technique [5]. The technique causes less damage to the integrity of chest wall, eliminate compression of the intercostal nerves with the use of a poking thoracoscope, and reduces local pain associated with the postoperative incision [6, 7].

In 2022, an estimated 1,918,030 new cancer cases and 609,360 cancer-related fatalities were reported in the United States, including approximately 350 deaths per day from lung cancer, making it the leading cause of cancer-related death[8]. With the development of diagnosis and treatment of pulmonary nodules, lung cancer presents a younger trend [9] and the rapid rehabilitation surgery concept has been ingrained into the routines of people. Therefore, most thoracic surgeons may focus on various aspects, including hilar and lung segmental anatomy, surgical techniques, endoscopic instruments, and thoracoscopy surgery, with the aim of improving the capability of surgery. Ignoring surgical incision healing is also important in the outcome and the patient aesthetic requirements.

However, considering that there is only one incision, the chest tube is inserted into the thoracic cavity after which an incision suture is formed. Patient activity, abrasion of the drainage tube, fat liquefaction, diabetes, steroids user, chronic kidney/liver disease and incision leakage may increase the possibility of delayed healing after removal of the tube. Generally, delayed healing of the incision, secondary debridement and suture, and scar hyperplasia may occur following chest tube removal. Currently, there is no standard surgical method for effective placement of the chest tube. The conventional methods of intermittent suture and chest tube fixation often increase the formation of hypertrophic scar in postoperative incisions, which does not promote rapid recovery. In the demix suture procedure, appropriate tension, skin temperature, tissue swelling, and adequate healing of the incision skin site are critical to incision healing. Herein, we improved the drainage tube suture-fixation approach which can be potentially applied in many clinical settings.

### Methods

This retrospective study analyzed the clinical characteristics data of 116 patients who underwent U-VATS to treat lung bullae or pulmonary nodules between October 2019 and October 2021 in Zhengzhou People’s Hospital. All the surgical procedures were successfully performed by the same surgical group. Patients were divided into two groups based on different suture techniques i.e., 72 patients who used improved suture methods (active group) and 44 patients who used traditional suture methods (control group). Signed informed consent form was obtained from all participants before the study and ethical approval was provided by the ethics committee of the Zhengzhou People’s Hospital (20,220,124).

Inclusion criteria: (1) no clear surgical contraindications in the preoperative examination; (2) successfully completed with U-VATS; (3) a thoracic drainage tube (20 F) was placed at the dorsal part of the chest through the incision, and a 10 F drainage tube was placed percutaneously through the 7th intercostal space on the posterior axillary line; (4) patients with elective surgery.

Exclusion criteria: (1) patients in the poor physical condition and unable to tolerate the operation; (2) conversion to multiport VATS or thoracotomy surgery intraoperatively; (3) the drainage tube repositioned without going through the surgical incision; (4) second operation through a similar incision or emergency operation.

### Surgical approach

All patients were given general anesthesia with double-lumen endotracheal intubation. The incision was located between the 4th or 5th rib of the midaxillary line and the front armpit of the surgical incision, with a length of about 3 cm. An incision was made into the skin and subcutaneous tissue, followed by the placement of the protective sleeve of incision (Changzhou Haida Medical Instruments Co., Ltd., disposable incision retractor, HRB-70/70 − 35/25). The procedure was completed using a 10 mm 30° lens with the surgeon located on the ventral side and the camera-holder assistant located at the back of the patient. After the operation, a 20 F drainage tube (Guangdong Xianlai Medical Instrument Co., Ltd., disposable multifunctional drainage tube, S10B) was placed on the dorsal part of the incision, and a single-chamber thoracic drainage bottle (Suzhou New District Ben Q Polymer Medical Instrument Co., Ltd., disposable thoracic drainage device, water-sealed, single-chamber type) was attached. Subsequently, a 10 F drainage tube was percutaneously placed between the mid-axillary line and the 7th intercostal space of the posterior axillary line below the incision (Shenzhen Cooper Technology Development Co., Ltd., disposable drainage catheter and accessories, DC-1025), after which the anti-reflux drainage bag was connected (Coloplast Medical Products Co., Ltd., disposable drainage bag, 1030). Finally, the repair was completed, two chest drainage tubes were positioned, and the chest wall was closed, thereby ending the procedure.

### Tube suture-fixation method

**Fig. 1:**
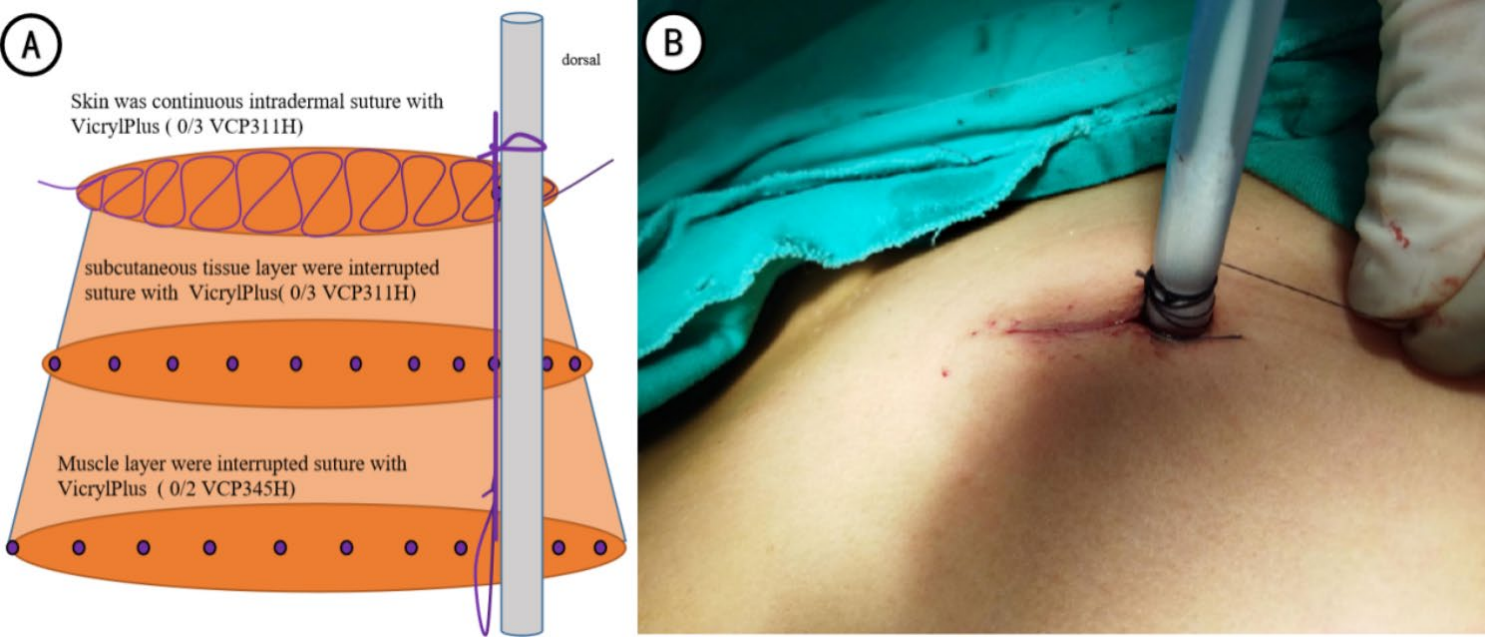
Schematic presentation and effect plot of the modified suture-fixation technique (A: Schematic presentation; B: effect plot)

In the active group, the muscle layer was interrupted by suturing with Vicryl Plus (2/0 VCP345H). First, a needle was sutured at the ventral subcutaneous tissue close to the drainage tube as the fixation line of the 20 F drainage tube. The tube was then fixed out of the skin after subcutaneous knotting was passed through the entire layer of the skin. The muscle layer was sutured, and the subcutaneous tissue layer was sutured intermittently with Vicryl Plus (3/0 VCP311H), carefully to ensure that there was no dead space and suitable tension under the skin. Thereafter, the wound was closed starting at the dorsal side 1 cm, away from the edge of the incision with Vicryl Plus (3/0 VCP311H) and left long enough to re-tighten after the removing of the chest tube in the dorsal side. Similar to the subcutaneous tissue suture technique, the needle was placed horizontally through the subcutaneous tissue by passing through the opposite sides of the wound. Notably, the suture continued around the chest tube until the needle reached the other end of the tube. Eventually, the ventral side of the drainage tube was knotted to separate the drainage tube and the remaining incision (Fig. 1); the remaining wounds were closed using knotless sutures(Video 1).

**Fig. 2:**
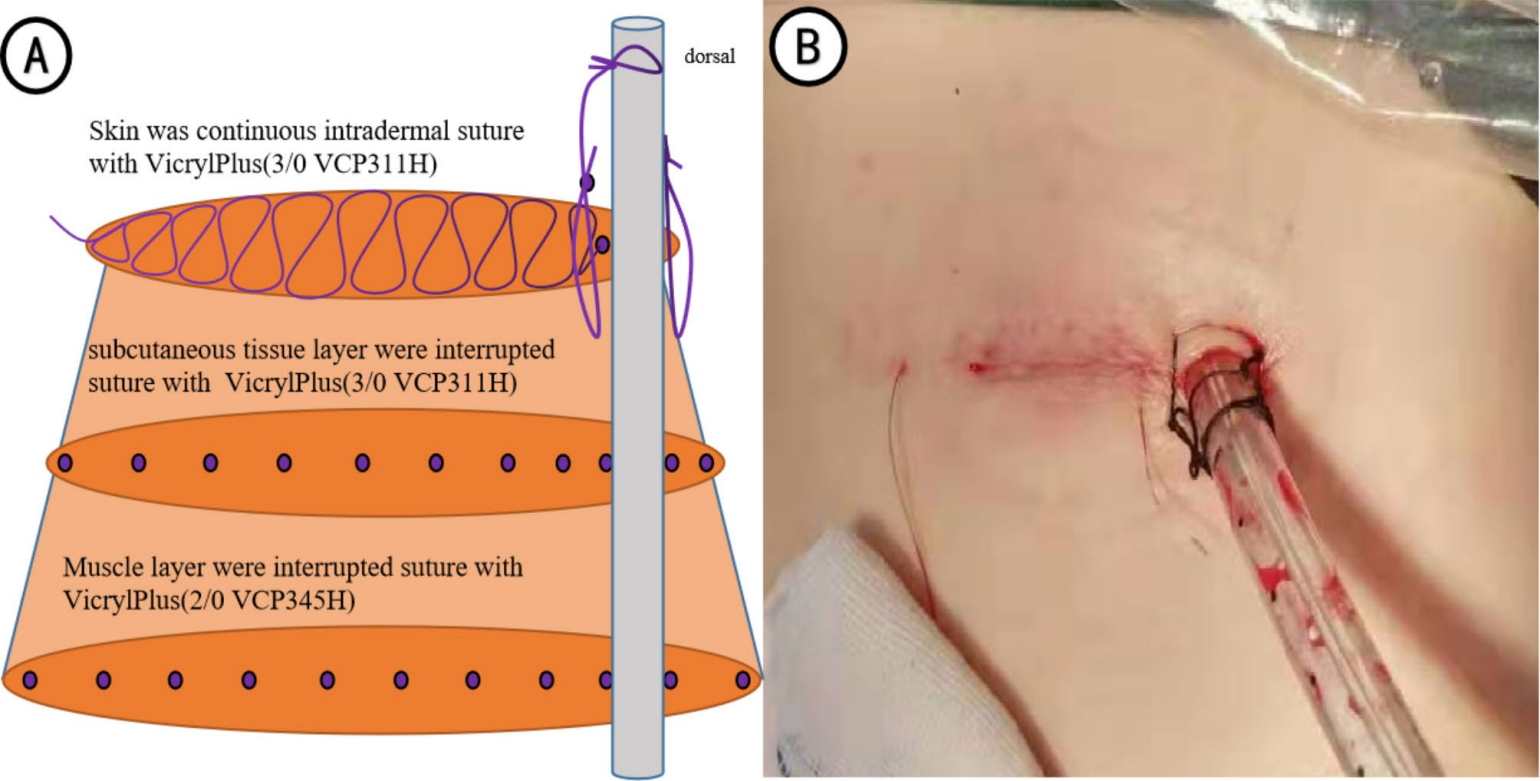
Schematic presentation and effect plot of traditional suture-fixation methods (A: Schematic presentation; B: effect plot)

In the control group, the same suture method was also used to close the muscle, subcutaneous tissue, and skin, except the drainage tube side. The “U-shaped” or “8-shaped” suture line [Vicryl Plus (2/0 VCP345H)] was reserved to knot and close the hole after removing the drainage tube, or the skin was sutured again at the time of removing the drainage tube. Notably, another line externally fixed the tube (Fig. 2).

### Secondary fixation of drainage tube and post-treatment of chest tube removal

**Fig. 3:**
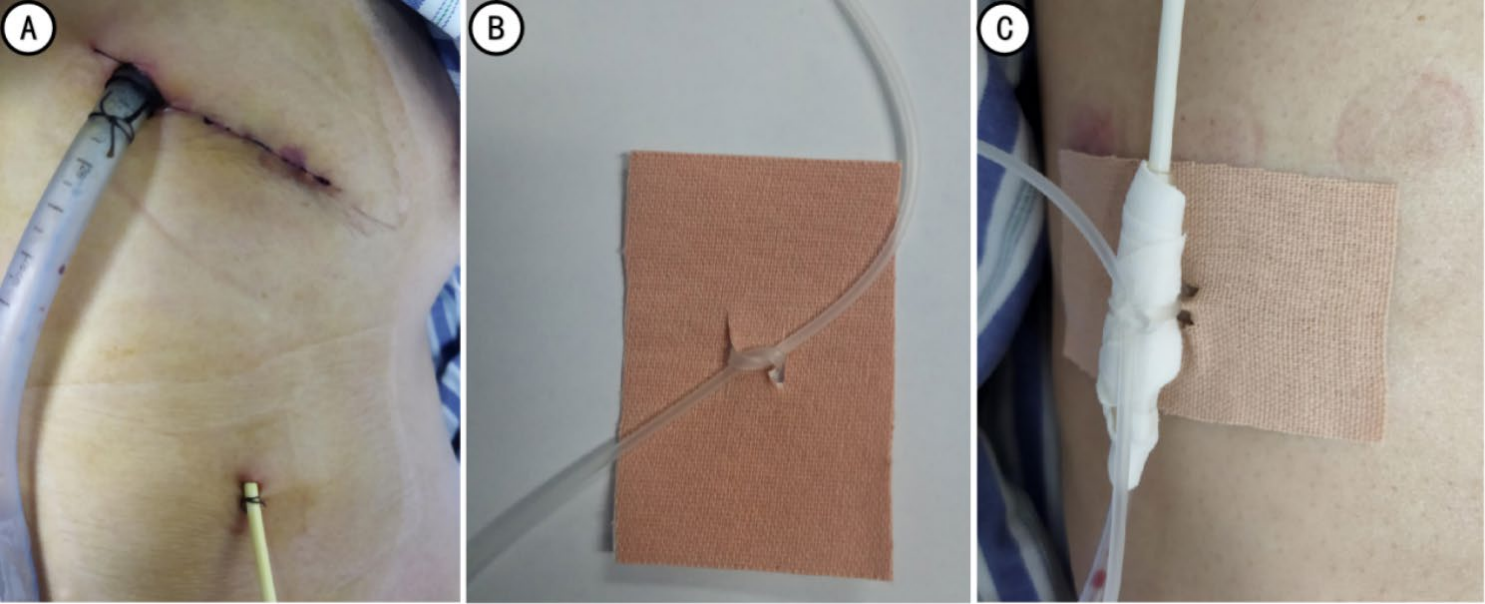
Chest tube indwelling and secondary fixation (A: chest tube; B, C: 3 M tape cutting method and secondary fixation of the chest tube)

Once the patient was returned to the ward, a 3 M tape was used to perform secondary fixation of the thoracic drainage tube to prevent unplanned secondary chest tube removal (Fig. 3). No unplanned chest tube removal occurred among the 116 patients during this period. On the seconding morning after operation, chest radiographs were taken beside the ward bed, and patients intensity and mode of activity, including getting out of bed and inflating a balloon to promote recovery, were determined based on inspection results.

The tube was only removed when the patient was able to deeply inhale and hold, the drainage number was less than 150 mL per 24 h with a clear color, the chest radiograph displayed good ipsilateral lung re-expansion without air leakage. The other drainage tube was kept smooth to relieve the associated pain and enable the patient to resume normal activities. After removing the larger-sized chest tube, the secured thread was pulled forward to tighten the suture and covered in the sterile dressing. The wound was then sealed with a zipper. The leftover thread was cut off after one day and nothing was left over at the scar of the chest tube site.

In the control group, the drainage tube was closed by directly knotting the reserved line or suturing it. In both groups, Vaseline and sterile dressings were applied.

### Follow-up and evaluation indexes

**Fig. 4:**
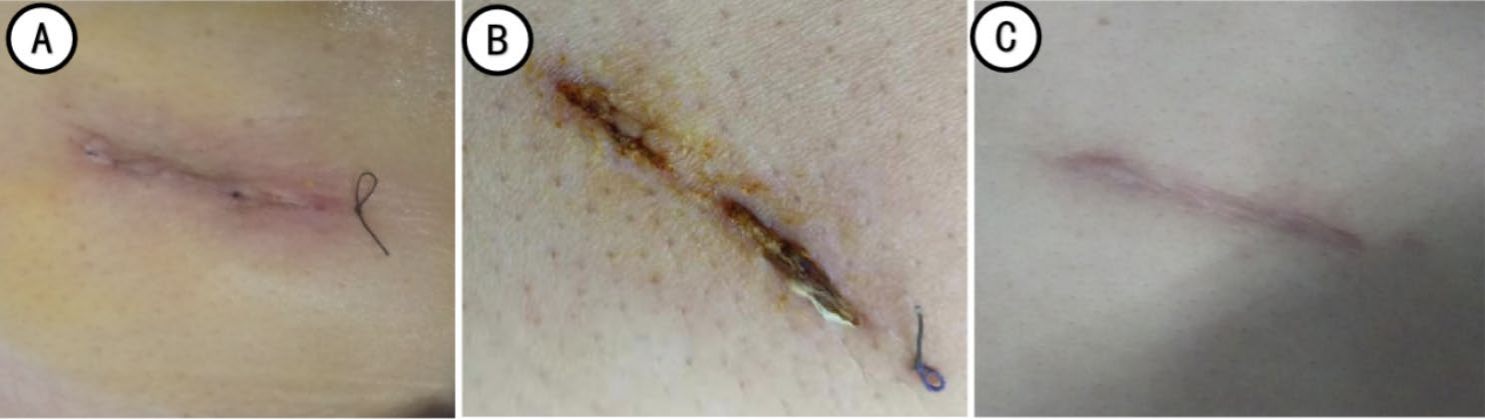
Incision and healing in the active group (A: the next day after chest tube removal; B: 10 days after chest tube removal; C: one month after surgery)

**Fig. 5:**
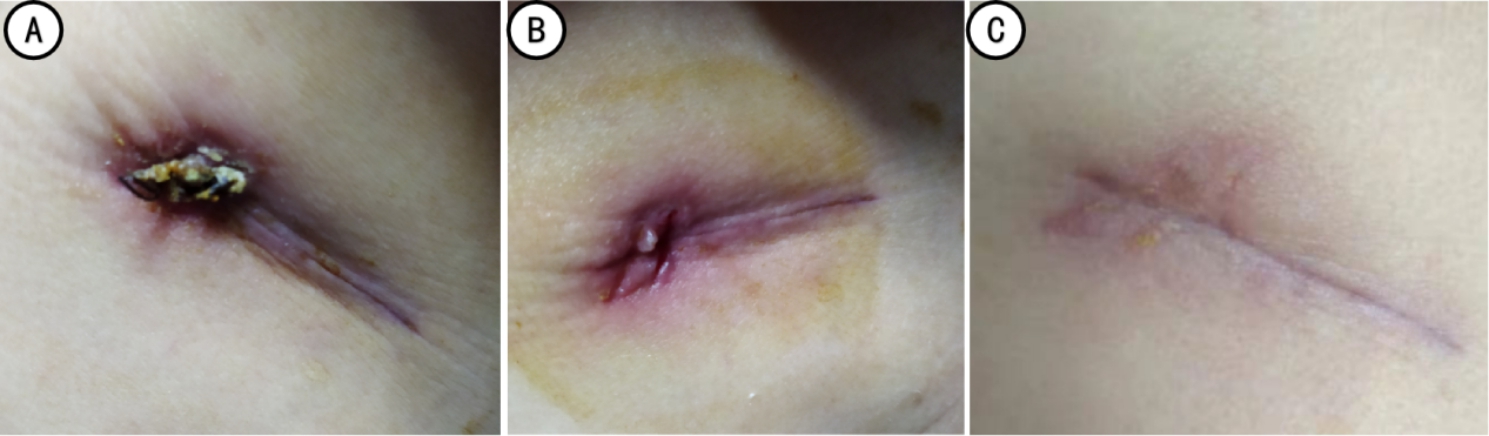
Incision and good healing in the control group (A: Before removing the sutures; B: After removing the sutures; C: One month after operation)

**Fig. 6:**
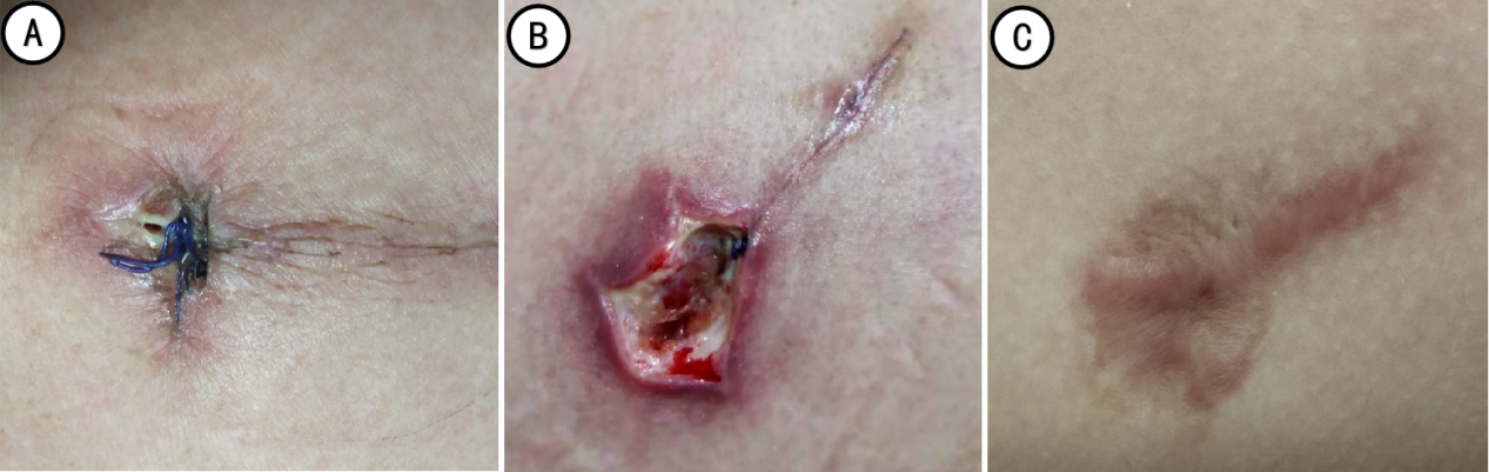
Incision and poor healing in the control group (A: the next day after chest tube removal; B: Second debridement after poor healing of incision; C: one month after operation)

The pain score was observed one day after the operation. The indwelling time of the larger-sized chest tube, removal time, wound healing grade at the discharged day, and the wound scar satisfaction were observed one month after the operation (Figs. 4, 5 and 6).

A patient-controlled analgesic pump was routinely applied on the day of surgery. The pain was assessed once a day after the operation using of a visual analog scale (VAS, vertical numerical scale ranging from 0 to 10 marked off in units of 1 point, with 0 score indicating no pain and 10 points indicating the worst possible pain. 1~3 points indicated mild pain; 4~6 points indicated moderate pain; and 7~10 points indicated severe pain). There was interference due to postoperative chest tube irritation, coughing, and post-exercise pain; therefore, the patient was asked to indicate the pain index in the steady state. The development of subcutaneous emphysema was rare during the first postoperative day.

The total time from disinfection to completion of dressing when the drainage tube was removed was recorded as the chest tube removal time.

**Fig. 7:**
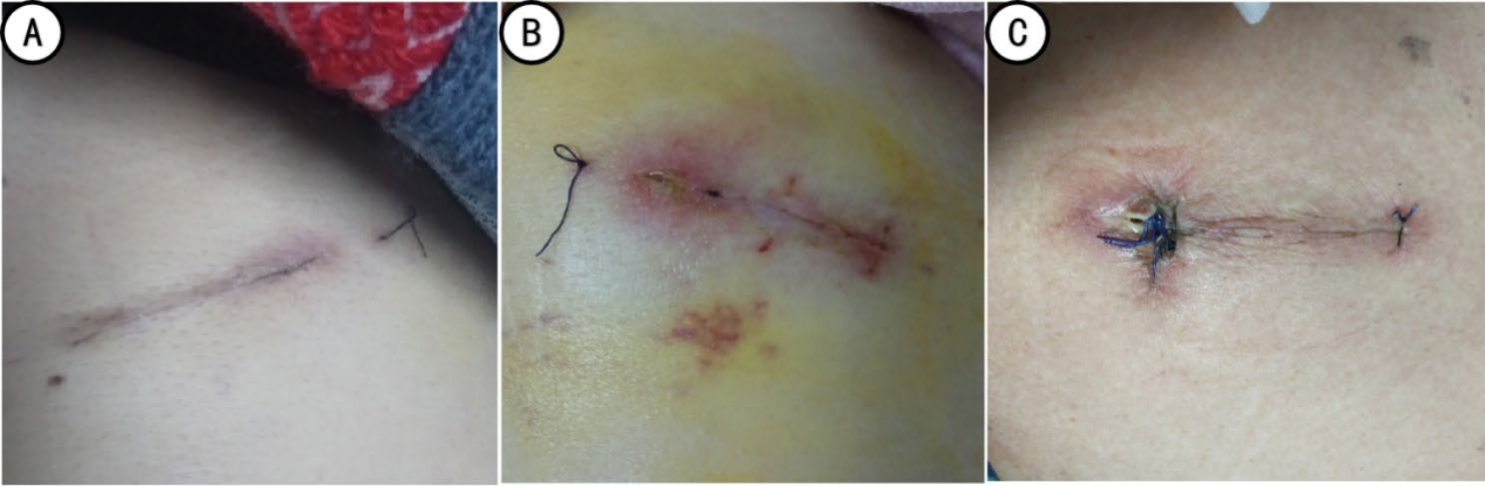
The incision healing grades (A: grade A, B: grade B, C: grade C)

The incision healing included incision leakage, secondary sutures, and wound infection. The healing was classified into grades A, B, or C based on the incision healing at the time of discharge (Fig. 7). Grade A healing represents excellent healing without any adverse reactions; grade B healing refers to poor healing with inflammatory reactions, including redness, induration, hematoma, and effusion, but without purulent; grade C healing indicates that the incision was purulent and needed debridement and drainage. Generally, sutures were removed 10~14 days after removing the thoracic drainage tube based on incision healing. The control group sutures were removed normally, whereas the sutures of active group were disinfected and cut off the skin; the remaining sutures subcutaneously were absorbed after a few days.

The Patient Scar Assessment Questionnaire (PASQ) was used to assess scar satisfaction by patients one month after surgery. According to the symptom, vascularization, pigmentation, thickness, relief, and scar pliability, incision scar satisfaction was divided into three responses i.e., 1 = very satisfied; 2 = satisfied; and 3 = dissatisfied. “Very satisfied” means that the incision had healed well with a slightly scar; “satisfied” refers to the incision having a small amount of exudation, delayed healing within three days, with a moderate scar; and “dissatisfied” indicates that the incision was purulent, the incision had not healed after changing the dressing for more than 2 weeks, or apparent scar hyperplasia and pigmentation. Incision scar satisfaction = (the number of patients with very satisfied + number of patients with satisfied)/the total number of patients ×100%.

### Statistical analyses

All statistical analyses were performed using SPSS version 23.0 software (SPSS, Inc., Chicago, IL, USA). Data were expressed as the mean ± standard deviation. Independent sample t-tests were used to compare the differences of parameter variables. The count data was expressed as rate, and the comparison between groups was performed by χ^2^ test or Fisher’s exact test. A *P*-value less than 0.05 was considered statistically significant.

## Results

U-VATS was successfully performed in all 116 patients, including 41 men and 75 women, with an average age of 55.78 ± 12.17 years. The participants included 73 patients who underwent limited lung resection surgical methods [wedge (included lung bullae and pulmonary nodules resection), segmentectomy], 25 patients who underwent pulmonary lobectomy, and 18 patients who underwent greater surgery (included lobe + limited lung resection and multi-site limited resection). The median indwelling time of the larger-sized chest drainage tube was 4 days, whereas the length of hospital stay was 7.34 ± 1.71 days. The intensity of pain one day after operation was mostly moderate. The incision sutured again or knotting with the reserved sutures after removing the drainage tube was avoided in the active group, so the chest tube removal time of active group was a great shorter than that in the control group, and a reduction in the chest tube was removed period would avoid patient inconvenience and improve satisfaction. The surgical incision had healed without any complications in the vast majority of cases. However, the incision healing grade and incision scar satisfaction was more lifted appearance than control group. Table 1 comprehensively shows the clinical characteristics of the patients.

**Table 1:** Clinical characteristics

Characteristics	Mean ± SD/number (%)/mean			t/χ^2^/Z value	*P-value*
	active group	control group	Total		
**Gender**	72	44	116	1.905	0.167
male	22	19	41		
female	50	25	75		
**Age/(years)**	54.61 ± 10.49	57.70 ± 14.43	55.78 ± 12.17	1.333	0.185
**surgical methods**				4.940	0.085
Limited lung resection	49	24	73		
wedge	44	22	66		
segmentectomy	5	2	7		
Pulmonary lobectomy	16	9	25		
Greater resection	7	11	18		
**indwelling time of the larger-sized chest drainage tube/(days)**	4(3,4.75)2–13	4(4,5)3–18	4(3,5)	-1.949	0.051
**pain score**				0.151	0.927
mild pain	2	1	3		
moderate pain	69	42	111		
severe pain	1	1	2		
**chest tube removal time**	20(19,21.75)15–35	50(48,51.75)46–54	22(19.25,44.75)	-9.044	< 0.001
**wound healing grade**				6.816	0.033
A	70	37	107		
B	1	5	6		
C	1	2	3		
**Length of stay/ (days)**	7.22 ± 1.29	7.52 ± 2.25	7.34 ± 1.71	0.916	0.362
**Incision scar satisfaction**	98.61%	86.67%	95.7%	63.821	< 0.001
very satisfied	68	10	78		
satisfied	3	28	31		
dissatisfied	1	6	7		

There were no significant differences in clinical features, surgical methods, chest tube indwelling time, and postoperative pain scores between the two groups (*P* > 0.05). However, a significant difference was identified in the chest tube removal time, incision healing grade, and incision scar satisfaction (*P* < 0.05) were better in the active group was superior to the control group.

## Discussion

Initially described in 2004 by Rocco [10] for a wedge resection, the U-VATS technique is appealing to most surgeons because it involves potentially less postoperative pain and is responsible for higher patient satisfaction regarding the number of incisions. Gonzalez [11] first used the technique for thoracoscopic lobectomy; as a result, its application has been broadened given the increased experience. Over the years, the U-VATS technique has been applied to various types of operation, including lobectomy, segmentectomy, sleeve resection of lung surgery [12–16], and mediastinal tumor surgery, including thymectomy and esophagectomy [17]. After a thoracic operation, the chest drainage is routinely placed with an underwater seal. However, the position at which the tube is placed differs between the center and posterior edge of the incision. We recently placed on the dorsal part of the incision.

Furthermore, the suture method, nutritional status, drainage tube diameter, and chest tube indwelling time have varying effects on wound healing. At present, limited studies have investigated the effect of the abdominal drainage tube e.g., the indwelling drainage tube effect on postoperative recovery after laparoscopic appendicitis [18, 19]. Limited studies, however, have explored the effect of the thoracic drainage tube. This study aims to explore a modified suture-fixation technique, geared towards improving the clinical application.

A suture is important in maintaining moderate tension, thereby ensuring adequate healing of the incision site. The ideal surgical suture should meet the following requirements: moderate tension, precise hemostasis, no dead space, and no permanent or only a few suture marks. Despite the many types of sutures, it is important to select suture materials matching the tension and healing speed of the tissue. After removing the chest drainage tube, the wound usually takes approximately 10~14 days to heal. The Vicryl Plus is a type of absorbable glycan lactic acid suture, with a tensile strength of about 75% when sutured for 14 days and approximately 25% when sutured for 28 days. The suture is completely absorbed after 56–70 days. Several studies indicate that the suture yields satisfactory results in clinical practice [20–22]. The clinical efficacy and suitability of knotless barbed absorbable sutures have long been reported with remarkable benefits [23–27]. However, the Vicryl Plus material could yield better clinical outcomes due to the hard texture and the cost.

With the improvement of the popularization of absorbable sutures and conditions for beauty, there is a rapid rise in surgeries including “small thyroid low suprasternal incisions and circular areola incisions for implantation of prostheses” in the Department of thyroid gland and breast as well as plastic surgery, “concealed incisions in the cavity” in the Department of Otolaryngology, and “single port laparoscopy, anastomosis, and specimen collection” in the Department of Gastrointestinal Surgery and Obstetrics and Gynecology. Therefore, surgeons have focused on minimally invasive and cosmetic requirements. Thoracic surgery is mostly categorized in grade three or four operations, where the trauma is relatively larger. Noteworthy, the healing of the surgical incision intuitively indicates the mental stress of the patient and promotes rapid recovery.

However, cases of delayed healing or even poor healing have been reported after removing the tube, substantially increasing the mental burden and extending the recovery time. This is unlike the concept of rapid recovery. In this regard, the present study evaluated a modified suture method that can properly fix the chest tube, accelerate incision healing, and increase the beauty of the incision simultaneously.

After thoracic surgery, proper indwelling and adequate drainage of the tube contribute to early postoperative getting out of bed and reducing the rate of postoperative complications, hence an important part of perioperative treatment and rapid recovery.

The currently available drainage tube fixation methods and post-chest tube removal treatment approaches involve ligation of extracutaneous sutures, followed by ligation and fixation again with sutures approximately 1 cm above the drainage tube out of the skin. After removing the drainage tube, the suture can be immediately ligated when the “U-shaped” or “8-shaped” suture is reserved during the operation. A re-suture under local anesthesia is necessary without a reserved thread. After closing the wound, it is covered with Vaseline gauze and multi-layer dressing to promote healing. Although research on the fixation of the drainage tube has been documented, most of these tubes are thin. At present, most of the used drainage tubes are larger-sized or both larger-sized, and ultrafine tubes are used simultaneously to promote activities of thoracic hemorrhage and rapid changes in the condition after thoracic surgery. This study investigated a larger-sized tube suture-fixation method. After a certain period of exploration and accumulation, this method, combined with a 3 M tape for secondary fixation, yielded the desired effect on the fixation of the chest drainage tube. Results revealed that none of the patients in this group had unplanned chest tube removal. Besides, the chest tube removal time wound healing grade, and incision scar satisfaction was significantly different unlike that of ordinary suture methods in the same period, suggesting that it merits clinical promotion.

Our experience is as follows:

1. Given that U-VATS incisions are usually like “trapezoidal”, skin incisions were small, whereas subcutaneous and muscle layer incisions were large. Therefore, since the focus should be channeled towards the suture of the subcutaneous and muscle layers on both sides of the wound to prevent leakage near the drainage tube, we recommended an interrupted sutures method.
2. After suturing the muscular layers on both sides of the drainage tube, the drainage tube was unable to slide up and down.
3. The muscle layer was sutured with Vicryl Plus (2/0 VCP345H). The suture was left on the ventral side of the drainage tube and used as the drainage tube fixation line. After several ligations, the drainage tube was ligated approximately 1 cm outside the skin.
4. The subcutaneous tissue was sutured using Vicryl Plus (3/0 VCP311H), and the skin was sutured from the dorsal side to the ventral side by an intradermal suture method. The needle was inserted approximately 1 cm outside the incision at the side of the drainage tube (the dorsal side), and approximately 5 cm was reserved outside the skin as a reserved line for removing the tube.
5. The needle was placed horizontally through the subcutaneous tissue by passing through the opposite sides of the wound similar to the continuous subcutaneous suture technique. A knot was tied on the inside of the drainage tube and the incision to prevent leakage or poor healing near the drainage tube opening from spreading to the entire incision. The suture continues around the chest tube until the needle reached the other end of the incision.
6. When cutting off the bundling drainage line, the end should be left into the subcutaneous tissue to facilitate suture absorption and avoid cutting the intradermal suture. When the tube is removed, the Vaseline gauze is compressed, the reserved suture was simultaneously tightened, and then the wound was covered with multiple dressings. Eventually, the wound was sealed with a zipper, and the suture was temporarily covered in the dressings.
7. In the short term after chest tube removal, the patient was instructed to press the chest tube removal site when coughing, and avoid excessive lifting of the ipsilateral arm to increase the tension of the incision simultaneously.
8. After one day, the sutures were pulled and knotted outside the skin, before cutting the excess sutures. After 10~14 days, sutures were removed based on the healing of the incision.

Our team often uses an ultrafine chest tube (10 F) combined with a traditional larger-sized tube (20 F) after pulmonary U-VATS. Nonetheless, a larger-sized tube was often placed in the incision, increasing the risk of poor wound healing. With the deepening concept of rapid rehabilitation and the development of thoracoscopy technology as well as Subxiphoid uniportal VATS [28, 29], the use of two 10 F pigtail tubes instead of the traditional larger-sized tube [30], as well as exploration of tubeless thoracoscopic surgery without endotracheal intubation and no chest drainage tube after surgery, has proven to be safe and feasible in a specific selected patient population [31–35]. However, this approach has limitations. First, it is still too early to be an alternative to routine surgical procedures and cannot be applied to wider populations [36]. This study was inspired by several explorations of suture technology and drainage tube placement [17, 37–40].

Given that this is a preliminary retrospective study, it has compelling limitations, including the retrospective nature and subjective bias, differences caused by different group members, no record of duration for each suture, and inadequate follow-up period. Additionally, we excluded patient factors in the analysis, including obesity, diabetes, steroid user, chronic kidney/liver disease, etc. Although the maximum indwelling time of the larger-sized chest drainage tube was 13 days in the modified method, it can significantly increase with more experience. Therefore, additional scar assessment is necessary.

## Conclusion

In conclusion, the thoracic drainage tube modified suture technique and chest tube fixation method described in this work are safe and effective with substantial cosmetic outcome. Moreover, the technique is a key component for the enhanced recovery surgery (ERAS) of thoracic surgery patients. We believe that this modified chest tube suture-fixation technique will be used in more patients.

## Electronic supplementary material

Below is the link to the electronic supplementary material.

Video 1: Experience of a modified chest tube suture-fixation technique in uniportal thoracoscopic pulmonary resection.

## Data Availability

The datasets used and/or analyzed during the current study are avaliable from the corresponding author on reasonable request.

## Abbreviations

U-VATS: uniportal video-assisted thoracic surgery
PASQ: The Patient Scar Assessment Questionnaire
ERAS: enhanced recovery surgery.
